# Iron in Cachexia

**Published:** 1847-01

**Authors:** 


### SELECTIONS

FROM

### AMERICAN AND FOREIGN JOURNALS.

---

*Iron in Cachexia.*—Iron is an agent almost exclusively employed in the treatment of cachexia, and its use has been in part recommended on the ground of the deficiency of its normal proportion in the blood. It is believed that the administration of ferruginous preparations may restore the normal quantity of the metal to this fluid, and on this ground the most soluble preparations are recommended, and, according to M. Mialhe, those chiefly should be chosen which are susceptible of decomposition by the alkalis of the blood.

M. Beau doubts the correctness of this view of the direct agency of iron, although the benefit of the substance is incontestible. The iron of the blood is contained only in its globules, and there will be more or less of it as these globules prevail. To cure hydræmia, therefore, and augment the proportion of globules, it can never suffice to introduce one element of the globule only. The diminished proportion of globules is maintained by the defective condition of the digestive functions, and iron acts only by restoring these to their integrity. In fact we daily witness cases of chlorosis treated by iron in its most soluble forms without any success whatever—the digestive organs in such cases being in a condition not admitting of their benefitting by its agency. The iron is abundantly absorbed into the blood, and yet the globule is not constituted. On the other hand, when the metal acts beneficially, the earliest effect it produces is upon the digestive organs. We see other patients again recover under the use of aloes, change of air, or the removal of moral causes, &c., without ever taking iron at all, or after abandoning it as useless—the dyspepsia having yielded to other means after the iron had failed in relieving it. Our primary indication then must be to attack *the cause which produces the dyspepsia..* M. Trousseau has already protested against this indiscriminate treatment of the cachexiæ by iron; and, in fact, we can only re-constitute the blood by re-establishing the digestive functions, and removing causes which operate injuriously upon them. It is not by the direct agency of this medicine, but by the greater amount of aliment it enables the digestive organs to master, that iron is useful; and cachexia induced by mere hæmorrhage may be at once removed by the rapid administration of aliment alone.—*Medico. Chir. Rev.*

